# Exploring meanings of illness causation among those severely affected by multiple sclerosis: a comparative qualitative study of Black Caribbean and White British people

**DOI:** 10.1186/s12904-015-0017-z

**Published:** 2015-04-19

**Authors:** Jonathan Koffman, Cassie Goddard, Wei Gao, Diana Jackson, Pauline Shaw, Rachel Burman, Irene J Higginson, Eli Silber

**Affiliations:** King’s College London, Cicely Saunders Institute, Department of Palliative Care, Policy & Rehabilitation, Bessemer Road, London, SE5 9PJ UK; Department of Neurology, King’s College Hospital NHS Foundation Trust, SE5 9RS London, UK

**Keywords:** Multiple sclerosis, Culture, Ethnicity, Palliative care, Neurology, Attributions

## Abstract

**Background:**

Illness attributions, particularly for those living with life limiting illnesses, are associated with emotional adjustment or psychological distress. Few studies have examined attributions among people severely affected by multiple sclerosis (PwMS), and specifically among from diverse communities. This study aimed to explore and compare the presence and construction of meanings among Black Caribbean and White British PwMS.

**Methods:**

Cross sectional qualitative interviews were conducted among Black Caribbean (BC) and White British (WB) PwMS with an EDSS of ≥6.0 (severe disease). Data were analysed using the framework approach.

**Results:**

15 BC and 15 WB PwMS were interviewed. Attributions were complex with most PwMS reporting multiple explanations. Uncertainty, represents the first theme surrounding the aetiology of MS where participants constantly rehearsed the “why me?” question in relation to their illness, a number expressing considerable frustration. The second theme, ‘logical and scientific’, was voiced more often by WB PwMS and accounts for a range of genetic/viral influences, stress, environmental and lifestyle factors. Third, the ‘supernatural’ illness attribution theme departs from a biomedical perspective and was reported often among BC PwMS. This theme included the sub-categories of tests of faith and divine punishment, a view although exclusive to BC participants but was sometimes in conflict with notions of modernity.

**Conclusion:**

Our findings identify evidence of cross-cultural and intra-group diversity in relation to MS causation. A greater professional awareness of the processes used by PwMS from diverse communities to make sense of their situation will enable health care professionals to facilitate effective support for those in their care and channel relevant psychosocial resources to them. This requires heightened skills in communication and cultural competency.

## Background

Multiple sclerosis (MS) is a common neurological disease causing disability in young adults. It is a highly unpredictable condition with a poorly understood aetiology and there is currently no cure [1]. Those affected by MS experience distressing symptoms which in the more advanced stages can include sensory and motor disturbances, fatigue, sexual dysfunction, pain, and bladder and bowel dysfunction [2], all of which have a profound effect on quality of life.

Attributional or explanatory models of illness encompass a person’s ideas about the nature of their problem and its cause. A number of studies within the sociological literature have qualitatively explored patients’ experiences of illness, particularly chronic illnesses. The ways people ‘make sense’ of these experiences have been shown to profoundly shake their life story [1-6]. Attributional activities also represent an attempt to restore a view of the world that is coherent and predictable [2]. They have been observed influencing all stages of the illness experience from decisions to seek medical advice and help, adherence to recommended treatment regimens, and adjustment to prognosis [3]. They have also been associated with positive physiological and psychological health outcomes [4,5].

### Multiple sclerosis and ethnicity

Geographical and ethnicity has been observed as being important modifiers of the global burden of MS; the prevalence of this disease is substantially higher among people living in the United Kingdom, the rest of Europe, North America, and lower among the indigenous people of Asia and Africa [6]. Genetic predisposition, and environmental factors such as the age of onset of Epstein Barr virus infections, and vitamin D exposure or a combination of all, may contribute to this variable prevalence [7]. Ethnicity is also thought to influence the clinical manifestations of MS; those of African origin have been shown to experience more aggressive disease and greater progression of disability [8]. Increasing evidence indicates a growing number of people from the second and subsequent generations of migrant populations to the UK developing MS [7].

Studies provide evidence of a possible relationship between MS-illness representations and adjustment outcomes [9,10] but have neglected patient-centred narratives on how people living with MS (PwMS) comprehend their illness. No research explored the meanings PwMS from black, Asian and minority ethnic communities attribute to MS and to what extent these are culturally shaped. This gap in knowledge should be addressed to reflect movements and relocations of populations resulting from increasing globalisation. Second, end-of-life care policy recommends that health and social care professionals invest in training to enhance knowledge and skills in cultural competency [11]. This is central to the success of ‘impeccable assessment’ of people living with progressive disease [12].

In this paper, we explore and compare Black Caribbean and White British people severely affected by MS. These attributions were examined in an embedded qualitative study that formed part of a wider population-based survey [13]. We chose to compare rather than focus on a single ethnic group because mono-ethnic group research can imply that they are ‘exotic’ [14] and by definition different compared to the majority population. The comparison may therefore develop a wider and more clinically generalisable understanding of commonalities and differences across ethnic groups.

## Methods

### Setting and participants

This study took place across two NHS hospital trusts and six primary care services serving a socially deprived geographical area with a population of 1.61 million people and one of the highest concentrations (25%) of Black Caribbean people in the country [15]. We also recruited via a voluntary sector organisation serving London’s BC community. Inclusion criteria included those who were: (i) 18+ years; (ii) confirmed MS diagnosis; (iii) Expanded Disability Status Scale (EDSS) score ≥6.0 (severe disease) [16] as assessed by a neurologist or a MS nurse; and (iv) self-identified as being WB or BC. Given the complexities and challenges surrounding the use of ethnicity as a variable [17] we asked all participants to self-validate their identify. In this respect the term ‘Black Caribbean’ is adopted in this paper to describe participants who identified themselves as having origins in the Caribbean and connections to Africa via historical antecedents. Patients were excluded if the clinical team believed they were unable to provide informed consent or would be unduly distressed by the study.

### Recruitment procedures

A subsample of eligible PwMS from both ethnic groups included in the population-based survey were purposefully identified to include a broad range of characteristics relevant to the study in terms of age, gender, MS type and disability level. Recruitment for each ethnic group continued until a point of theoretic saturation was reached [18]. Eligible PwMS were provided with a study participant information sheet and given 48 hours to consider their involvement in the study. Those who expressed an interest then contacted CG or JK to organise a convenient time and location (usually the participant’s home) for a face-to-face interview. Each participant provided written informed consent.

### Data collection

The interview was developed in collaboration with user participants from a London-based MS patient advocacy service for the BC community. We asked participants: “Tell me in your own words why you think you became ill”? Prompts were then used to elicit further information when required. All interviews were audio-recorded and lasted on average 77 minutes (20-120 minutes). Detailed field notes were recorded to expand impressions of the interview.

### Data analysis and trustworthiness

Interviews were transcribed verbatim, anonymised, and analysed first by CG using the framework approach [19]. To address issues of analytical rigor and trustworthiness a subset of transcripts were double-coded by JK. A re-iterant process of discussing areas of agreement and disagreement took place between CG and JK to achieve consensus. Alternative interpretations were incorporated in the analysis. The analysis was further tested during discussions with colleagues and meetings of the project advisory steering group. We also paid attention to non-confirmatory cases where emerging themes contradicted more common ideas. Participants’ ethnic identities are preceded by ‘BC’ (for Black Caribbean) and ‘WB’ (for White British). To preserve study participants’ identities all names throughout this paper have been pseudo-anonymised.

## Results

### Sample characteristics

Participants from the BC community were typically younger than their WB peers and had been living for a shorter period of time with their MS (BC median 9 years v/s WB median 18 years). While most BC participants were second generation, two participants had moved to the UK from Jamaica aged nine and twelve years (Table 1).

**Table 1:** **Characteristics of study participants**

	**Black Caribbean**	**White British**
	**(n =15)**	**(n =15)**
**Gender:**		
Male	5	5
Female	10	10
Mean age (range) Median age	46.6 (27–60) 48	56.9 (35–71) 57
**Country of birth:**		
UK	13	15
Caribbean	2	0
**EDSS score:**		
6.0	4	6
6.5	3	2
7.0	3	2
7.5	2	0
8.0	3	4
8.5	0	1
9.0	0	1
**MS type:**		
Primary progressive	6	5
Relapsing remitting	4	2
Secondary progressive	5	8
**MS duration:**		
Mean years since diagnosis (range)	9 years (10 months – 22 yrs)	18 years (4 – 47 yrs)
**Highest level of education attained:**		
Primary school	1	0
Secondary school (GCSE)	5	8
Secondary school (A-Level)	6	2
University/HE	3	5
**Living arrangements:**		
Owner occupied house/flat	5	7
Privately rented house/flat	0	1
House/flat from local authority	9	7
Sheltered accommodation	1	0
**Religion volunteered:**		
No religion	1	4
Christian	12	11
Other	2	0
**Current employment status:**		
Employed part-time	0	2
Unemployed	2	2
Retired due to age	0	4
Medically retired	9	6
Student	1	0
Housewife/husband	1	0
Other	2	1

All participants volunteered views on the meanings of their MS. We observed that the causal attributions provided by participants from both ethnic groups were complex, multi-dimensional, and in some instances co-existed together. Three central themes emerged from the interviews: uncertainty, logical and scientific, and supernatural explanations.

### Uncertainty

This theme refers to uncertainty surrounding the aetiology of MS. Whilst a number of participants spoke of rehearsing and then re-rehearsing the “why me?” question and possessed well-fashioned accounts of their MS causation, others remained unsure how to make sense of the inexplicable. Megan (not her real name), a 35-year old White British woman with relapsing remitting MS, was confused about possible causes of her illness:

*I’ve read a lot of opinions … of what other people think … scientists and so forth, but there seem to be so many of them that the bottom line would be they don’t actually seem to know . As a child I was quite sick with the chickenpox. And then some people say, oh it’s because you don’t get enough vitamin something or rather, whichever it is that’s in the sunshine… I was like “Yeah, whatever”… I just don’t know, it’s a completely misunderstood (laughing)* (WB53).

For a small number of participants, the interview represented the first opportunity where participants attempted to rehearse the question of MS causality. For example, Grace, a 57-year old Black Caribbean woman with primary progressive MS, reflected for a moment and then it became apparent that her unknowing troubled her, at least in relation to comprehending the consequences of MS and her mortality:

*I don’t know why I became ill really, but people become ill. I was fine, I was very active and then one day I found I couldn’t feel my leg. Then they diagnosed MS. Didn’t know what it was about. I’d heard the name but didn’t know how or what it involved. Didn’t know how it affected one. Didn’t know if it was a life or death thing. I just don’t know* (BC57).

This sense of unknowing was amplified by participants’ frustration with medical professionals who were unable to provide convincing explanations to account for their illness. Lillian, a 38-year-old Black Caribbean woman with secondary progressive MS typified this concern:

*What makes it worse is that doctors don’t even know where it comes from, how you get it … it’s not like cancer, you know what might have triggered that off. So it could be like, you know, lung cancer, maybe it’s drinking or smoking too much …and with MS it’s, you just don’t know why (BC 82).*

### ‘Logical and scientific’ explanations

The second main theme represents a range of ‘logical and scientific’ represented a body of rational and ‘common-sense’ illness attributions; it included views of those who believed their MS were due to genetic or medical influences (e.g. viral infection), environmental influences and lifestyle factors, bodily insults, and stress. More WB than BC participants attributed the origin of their MS as genetic or pre-empted by a medical insult. There was a tendency for those who held this view to endorse hereditary factors when discussing aetiology, with five of the fifteen WB PwMS interviewed reporting a familial history of the condition. This was highlighted by Olivia, a 69 year old WB female diagnosed with secondary progressive MS. She deliberated for a moment and then shared her belief that there was a possibility of a link between her own fate and that of her grandmother’s MS many decades before:

*I think I very probably inherited it from granny or someone in the family (WB 11).*

In contrast, other WB participants appeared to be less convinced about the roles these complex and elusive factors played in relation to MS. They were more resigned to their fate. For example, Anna, a 45 year old with primary progressive MS suggested:

*Well, that’s life. Yeah, that’s what I’ve always said, it could happen to anyone. They don’t know if it’s in your genes, or if it’s in your body makeup. I don’t think, once I found out it wasn’t hereditary, so I hadn’t given it to [daughter], cos it’s not hereditary, it’s no-one’s fault. That’s life. Get on with it! (WB 08).*

A number of participants endorsed the view that their environment, surroundings or lifestyle may have contributed to the onset of their illness. Participants from both groups questioned the impact of previous health behaviours. For example, diet or the use of illicit substances with onset of their MS. Michael, a 58-year-old WB man with secondary progressive MS had worked for many years as a plumber, and speculated that his exposure to harmful and toxic chemicals in his workplace played a critical part in the onset of his illness:

*…dealing with all the chemical stuff we used, like the chemicals to put in central heating systems and to drain it all out again – There was this (name of chemical) stuff that we was using. If you splashed it on your overalls it would form a white mark and then the next morning there’d be a big hole, you know! I just don’t know whether it was that* (WB 27).

The influence of current research and information available in the public domain also appeared to have influenced participants’ views on MS causation. For example, two WB participants referred to the influence of Vitamin D deficiency as playing a contributing role in the manifestation of MS in their lives. This was a credible catalyst they had either read about, or been informed of by health care professionals. Eleanor, a 57 year old with relapsing remitting MS, illustrates this sentiment:

*I came across something recently. It all goes back to vitamin D. That someone with MS is more likely to be born at the time, time of year that I was born. Which was April, because, the gestation period is through the winter, so they’re not getting very much vitamin D. And also the fact that my mother was so ashamed that she was pregnant with me that she didn’t go out hardly at all! (WB 22).*

Whilst some were quick to suggest their own health behaviours contributed to their illness, others found it quite impossible to make explicable why they had ended up ill, implying they had led blame-free lives. Liz, a 49-year-old WB woman with relapsing remitting MS, explained her reaction when first diagnosed:

*…I remember there was a lot of questions when I went to the doctor and said “Why me?” because I said “I’m not, I don’t, I’ve never touched drugs! I don’t drink. I’m very healthy”. So I couldn’t understand…. So there I was, I do all these healthy things, I couldn’t think why on earth me? (WB 32).*

Other study participants felt equally absolved from personal responsibility over events. This paradoxically appeared to help them accept their illness and locate a sense of calm. Granville, a 50-year-old BC gentleman diagnosed with primary progressive MS, best illustrates this. He reported:

*There’s no cause. It’s not that you’ve lived a wrong life, a bad life and you’ve been abusing your body to cause MS. In cancer you can say, “Yeah, I was smoking”, you can kick yourself but you can’t turn the clock back. But with MS, no one’s to say, ah you weren’t eating your fruits n veg when you were a kid, or you used to eat too much chocolate! No one can say that. But that’s the good thing about it. The nice thing about it is you didn’t cause it….So I can live with that* (BC 46).

A further subtheme represents bodily insults and specifically refers to accounts where BC PwMS attempted to interpret their illness through the lens of previous accidents, injuries or invasive medical procedures. These were either directly, or in combination with other insults, key triggers that led to their MS. This was best illustrated by Austin, a 48-year-old BC man with secondary progressive MS. He explained he connected his MS to an accident that preceded his diagnosis:

*I was in the wrong place at the wrong time. I tripped! A week later I start limping, and I thought what’s wrong? I had this specialist look at me and put me under the MRI scan. And they tell me I’ve got MS! And if it wasn’t for that trip, man, I wouldn’t be having no problems now* (BC 43).

Stress, a ‘logical or scientific’ explanation, refers to participants who believed their illness was a direct response to a psychological trigger. Some PwMS recounted historical dysfunction, often coupled with stressful and destructive relationships with others. Others laid blame at being overworked or catastrophic life events. Participants readily suggested that stress depleted their immune system and it was this that precipitated their illness. Marlene, a 60 year old BC woman diagnosed with a progressive form of the disease, described a series of seemingly unconnected traumatising and distressing life events which she was convinced contributed to the onset of her illness. The litany of insults included racism, bullying at school, her parents’ divorce and surviving foster care. She said:

*…I have a strong suspicion that MS is stress-related… I’ve noticed certain times of my life when social pressures have been such that I’ve been quite traumatised….where I’ve got distressed. I think it does something in the immune system that we don’t yet understand* (BC 18).

The accumulation of corrosive life events was also shared by Lydia, a 57-year-old WB woman with secondary progressive MS. She strongly suspected the relentless pressure associated with being a full-time caregiver for a loved one acted as a catalyst in triggering her MS. She said: …*she [friend] said that she wasn’t at all surprised that I got MS, because I’d had seven years of sleepless nights, living on my nerves. My body system had collapsed, and I guess I’ll go with that really *(WB 13).

### ‘Supernatural’ explanations

For some, perspectives on MS causation moved away from a biomedical perspective to one that resided within a ‘supernatural’ domain. We comprehend this domain as a manifestation or event, attributed to some force beyond scientific understanding or the laws of nature. This theme was populated by more BC than WB participants who often gravitated towards divine influences to make sense of the inexplicable reality of their illness. This supernatural theme comprised three subcategories: ‘my challenge, my test’, ‘punishment’, and ‘fate, destiny, or just bad luck’.

The belief that MS could be attributed to a ‘challenge’ or ‘test’ was a view held only by BC participants and was deeply embedded within their religious belief system. Some participants were quick to state that they firmly believed that God had specifically chosen them to experience MS believing it occupied a dimension for spiritual growth to help them gravitate to a more sanctified dimension. When expressed, participants stated MS was an opportunity to review some of life’s more challenging vicissitudes as positive life-affirming events that they willingly accommodated. Hazel, a 46-year-old BC woman with secondary progressive MS, stated:

*I do feel it’s a test, I really do. Because it’s something my mum said the night when I rang her and told her that I had MS. She said to me “Read your Bible”. And then she told me what chapter to read. I started reading it. For me, it made me feel a lot lighter. I didn’t feel like I had a burden on my shoulder. I felt like something, some entity had just taken it off my shoulders. So for me to have coped with it for this long, and I’m still bubbly and still outgoing. (But) I think it’s a test. It’s how I deal with it* (BC 15).

While some described enthusiastically greeting their divinely-provided test, others provided accounts that appeared to question the purpose of continuous tests that brutally reduced them. This painfully challenged their orthodoxy of an all-caring and benevolent God, and it hurt. Faith, a young 27-year-old BC woman with relapsing remitting MS who had lived alongside her condition for 11 years paused for a moment and then said:

*Everyone says Jesus and God love everyone and, and sometimes they’ll put you through things and let deal with it. Buts sometimes I think to myself, God don’t like me, he just don’t like me. That’s it! If he liked me he wouldn’t have let me go through. It’s like he wanted me to hurt - to go through so much and deal with it - there’s no light at the end of the tunnel to happiness. It’s been hell. (participant laughs). But I’m trying my hardest, harder than you know. I’m surviving. If Jesus is trying to test me, he’s also got a lot to answer for because he’s just like pushing my buttons to the extreme. I feel it’s getting too much* (BC 65).

The category of fate, destiny, or just bad luck was also specific to BC participants, a number of whom drew on biblical phrases to help convey their thoughts. They provide accounts where their MS was viewed an inevitable part of God’s life plan for them. Lillian was convinced that her lot was predetermined by God. Moreover she stated that it was impertinent to question the path she been chosen for:

*From the day you’re born God knows which direction you’re going to take. Your life is already mapped out. I just don’t think it’s up to you to ask* (BC 82).

Punishment represented the last supernatural theme and was characterised by wrongdoing that in some instances justified retribution. It was voiced by participants across both ethnic groups who either perceived their punishment as being justified, levelled at them personally or more widely at humankind. However, a small number of WB participants refused to blindly accept this meaning. Anthony, a 43-year-old with secondary progressive MS, exemplifies this sentiment. After sifting through his life he failed to successfully identify instances of profound wrongdoing or misdemeanour. For him, the punishment of MS was disproportionate and undeserved:

*It feels like I’m being punished for something … I wouldn’t wish on my worst enemy, you know, but I haven’t done anything to cause it, as far as I know* (WB 37).

Of the participants who chose to identify the source and outcome of their personal or group sin as being legitimate, the outcome was more easily accommodated. Hazel, a 46-year-old BC divorcee with secondary progressive MS stated matter-of-factly that her uncharitable thoughts and lack of devotion to God may have accounted for her illness:

*I did something bad. I thought bad thoughts. I cursed someone. I wished somebody dead. I wished the ex-husband dead! I hated him. I wished him dead. I want to like to chop him up and whatever. I don’t go to church. So I’m thinking probably it’s God’s way of, you know. God’s way of saying, oh let’s her out, keep her busy. It’s a punishment for me* (BC 15).

An important interpretation of punishment emerging from the analysis relates to how a number of BC participants choose to challenge an accepted orthodoxy of sin endorsed by some family, friends and community. Sin may have represented an explanation for some, but not all. For example, Hope, a 48-year-old BC woman with primary progressive MS provides an account where she chooses to reject mainstream thinking. Divine punishment has little currency for her:

*…with most African Caribbeans you’ve done something wrong to be sick like this; you ended up in a wheelchair… I don’t know if that’s like the same for English people. But it’s definitely a black cultural thing. Being amongst the black community, that’s what it’s like, and it’s very hard …I’m working with myself, if that makes sense, to not particularly care or take it on because what I’m thinking is it’s probably nothing to do with it what they’re thinking* (BC 79).

## Discussion

This study adds important new insights into the socio-cultural illness attributions held by BC and WB people severely affected by MS and makes a contribution to an emerging evidence base on the interface between ethnicity and health beliefs about progressive and advanced disease [20].

### Methodological reflections

There are limitations with our study that warrant consideration. First, the cross-sectional study design did not permit exploration of meanings over time. Illness attributions are not static constructs and may fluctuate from one time point to the next as a result of a multiplicity of social situations. It is also possible that meanings converge with one another to form clusters, which individually may be inconsistent with one another. Future research using prospective study designs would help to explore these phenomena.

Second, the method of data collection may affect the veracity of responses, particularly in sensitive areas. Here ‘racialised differences’ between research participant and interviewer may potentially influence what they are prepared to share in relation to their illness experience [21]. It has been suggested that people from minority groups may be reluctant to volunteer supernatural causes to researchers from white, middle-class backgrounds, who are likely to view such attributions as "superstitious" [22]. Matching interviewer and participant on characteristics such as ethnicity (CG and JK are WB), gender, age, religion, or experiences in relation to the research topic may establish rapport. Despite this concern, we were successful in recruiting, undertaking highly sensitive interviews across both ethnic groups, and eliciting meaningful accounts to understand participants’ MS illness attributions. Moreover, true ethnic matching is difficult since it addresses only one possible marker of social identity and is further complicated by suggestions that we are all subject to ‘social hybridity’ [23].

Third, this study did not aim to examine the relationship between illness attributions and patient centred health outcomes or coping responses. Whilst previous research among those with breast cancer [4], those with unexplained neurological symptoms [24], and those with multiple sclerosis [25] have attempted to examine predictors of clinical outcomes that included illness beliefs, these studies did not specifically focused on diverse communities. Future research should attempt to examine how culturally shaped illness attributions among those with multiple sclerosis influence illness cognitions and perceptions of control.

### The centrality of meaning making

There were some commonalities across ethnic groups in the ways causal attributions were constructed. First, uncertainty and MS were present among PwMS in both groups. Second, a number of participants in both groups also voiced ‘logical and scientific’ explanations, views that fitted with a biomedical model of disease causation. However, a number of important culturally specific dimensions were evident which have implications for the way diversity is serviced during the clinical encounter. Insights that were specific to Black Caribbean participants were often at variance with conventional, rational explanations; instead some polarised to more ‘supernatural’ explanations, often connected with deeply held religious beliefs.

The causes of MS are not well elucidated. Like many complex conditions, the disease is due to a variety of genetic and environmental factors, each of which is likely to contribute a small amount to the overall risks of developing the disease. This concern was magnified among participants where confusion was similarly evident. Some participants’ understandings were either attributional absent or involved confused speculation. These are not unsurprising observations and find correspondence with previous evidence that people living with illness often identify with multiple explanations which lack any scientific basis [26]. Whilst it has been suggested that people who relate to complex multiple attributions adapt more easily to their illness [27], Mishel and others have observed that ambiguity and uncertainty lead to more challenging comprehensions which generate stress and inhibit effective coping [28,29]. We observed this in relation to the perceived unpredictability of the illness and the stress some voiced against a backdrop of possible mortality.

### Meanings of illness and cultural patterning

The focus of logical and scientific’ explanations were prominent across both ethnic groups but differed in culturally important ways. Instead of gravitating towards views underscored by an established scientific orthodoxy, BC participants preferred instead to seek out their own rehearsed logic to substantiate and comprehend their situation. This might be related to the current reality that MS is a new disease in this community and is typically being a disease associated with Caucasians [7]. Examples included the possibility that MS results from ‘bodily insults’. This greater physical emphasis has been observed before among those living with cancer; one USA-study reported that African American women diagnosed with breast cancer believed physical insults including ‘blows to the breast’ were directly to blame for their condition. White women rarely endorsed this view [30]. Previous research has controversially suggested that African Americans sometimes articulate views that have less scientific credence than Caucasians, and that this may be associated with education level [31]. However, commonalities were noted, and the idea of stressful life events contributing to the onset of their condition was endorsed by BC and WB PwMS.

A number of MS attributions, and specifically the supernatural attributions, we observed support aspects of the “Meanings of Illness” theoretical framework developed by Lipowski for conceptualising how people cope with illness [32,33]. He proposed eight meanings of illness where patients respond differently to their situation, some viewing it positively as a challenge or test, whilst others negatively, for example as a punishment. This framework acknowledges that meanings may be shaped by inter-personal factors such as age, personality, values and beliefs and the likelihood of cure and prognosis.

We identify that BC participants referred to the influence of divine tests to help account for their MS causation, a view that was rarely endorsed among those who identified as being WB. These views correspond with Pargament and colleagues who refer to benevolent religious reappraisals as an attempt to redefine a stressor as having spiritual benefit and an opportunity to grow spiritually [34]. Research among church-going African Americans [35] and British Afro-Caribbeans [2] with cancer also notes that suffering during illness and at the end of life is noble. This position is in contrast with Charmaz’s research among a predominantly White American, female patient population with MS, heart disease and cancer. She notes: *‘The language of suffering severely debilitated people spoke was a language of loss. They seldom talked of gaining a heightened consciousness of the world, revelations about self or insight into human nature from their experiences’* [36].

We also identified attributions akin to a punishment. Faced with patients who hold this view, Lipowski states that health care professionals must engage in dialogue with patients to reappraise their situation and to seek alternative meanings associated with positive coping. It is here we propose the framework requires some reinterpretation. First, the notion of punishment may not apply in its current configuration. This is particularly so when it is suggested punishment leads to atonement, initiative, vigour previously absent before the onset of their illness, and perhaps recovery [32,33]. All participants in this study were severely affected by MS; the possibility of full recovery is not a reality. Our findings also suggest despite its distressing nature, punishment was not always perceived negatively, and in some instances accepted. This challenges Lipowski’s and others’ [4,37] notion of a single universally understood interpretation of this meaning. It is here health professionals are encouraged to engage with, and to attempt to modify, views of patients who voice maladaptive meanings. Importantly, whilst this meaning may have viewed as redemptive for some participants it was not a view held by all. Inter-generational points of friction were evident where traditional beliefs held tightly within the community were open to question. This important departure from accepted opinion may reflect MS as being an illness associated with younger people; a process of acculturation may be leading to a departure of more traditional values [38]. Second, Lipowski states the meanings of illness seemed to be most prevalent in the culture he developed the framework (Canada and the USA) where research using framework has focused principally on cancer and among White patient populations [4,39]. Consequently, the meanings and their interpretations may not apply readily to different groups located elsewhere.

## Conclusions

Multiple sclerosis is a life-changing encounter influenced by many factors including illness attributions, some of which are culturally and patterned. It is not enough to know how to care for patients with MS and their symptoms; health and social care professionals also need to care for them sensitively and holistically. When providing explanations, recommending therapies and in advanced care planning it is helpful to have an understanding as to the patient’s conception of their disease. Health education campaigns and the media bombard the public with messages that have the potential to change views about the origin of diseases and may have significant impact on how they conceptualise illness, including MS. Illness causes that attract much public attention typically include genetics, lifestyle, psychological factors, and environmental factors. Considerably less attention is focused on supernatural causes, yet we identify that these beliefs are graded by some as performing a central role in meaning-making. Moreover, they have potential to bring order to stressful life events associated with multiple losses.

Health care professionals have been criticised for patronising patients by ignoring their ‘illness narratives’ or health beliefs [40]. Therefore, we recommend health and social care professionals make use of narrative and biography to explore and to obtain a greater professional awareness of the ways culturally shaped illness attributions impact on how patients comprehend their illness and seek help. Moreover, when performing an assessment with patients from different backgrounds to their own, health and social care professionals should sensitively ask questions that go beyond a detailed description of symptoms. For example, they should facilitate opportunities for patients to express information about their illness that include the centrality of meanings shaped by religious beliefs, and how these alter their perceptions of illness and quality of life [41]. The importance of this also has implications for the channelling of limited psychosocial support. It may be counter-productive to focus resources on those who hold well-formulated culturally patterned notions about their illness, which, although distinct from biomedical conceptions, nevertheless, serve them positively in times of crisis. Faced with patients who hold these views, physicians have historically been urged to assist them in health promoting dialogue to reappraise their situation and to seek a meaning associated with positive coping [33]; this has been most apparent among those with breast or colorectal cancers [37,39,42]. However, our findings highlight that attributions which depart from logical or scientific foundations are not inevitably harmful to the individual. We therefore believe it may be more prudent to target support to those who are particularly troubled by poorly articulated attributions, or who occupy positions of clinical or cognitive uncertainty. Health care professionals should consider supporting these people develop new meaningful post-diagnosis identities in the context of their rapidly changing circumstances [43]. Interventions to support this approach include nurse-led and cognitive behavioural therapy (CBT), which appears to be successful in the early stages of MS [44,45], but has not been tested in more advanced illness or across ethnically diverse populations.

We are also mindful that illness attributions represent a continuum, which are fluid and open to reinterpretation, and which may also co-exist with one another. This implies that illness attributions and highly complex and may change during the career of a person’s MS. Detailed and sensitive exploration of these meanings may provide a useful screening approach to identify those at risk of reduced psychosocial functioning.

### Ethics approval

King's College Hospital Research Ethics Committee (South London REC Office (2) (ref: 10/H0808/43) and Research & Development Departments (Lambeth, Southwark, Lewisham & Bexley RDLSLBe545, Guy’s & St. Thomas’ RJ112/N042, and South London Healthcare SLHT/2010/UCSN/neuro/27).
